# Effects of a Brazilian cardioprotective diet and nuts on cardiometabolic parameters after myocardial infarction: study protocol for a randomized controlled clinical trial

**DOI:** 10.1186/s13063-021-05494-0

**Published:** 2021-09-01

**Authors:** Aline Marcadenti, Bernardete Weber, Angela Cristine Bersch-Ferreira, Rachel Helena Vieira Machado, Camila Ragne Torreglosa, Enilda Maria de Sousa Lara, Lucas Ribeiro da Silva, Renato Hideo Nakagawa Santos, Debora Harumi Kodama Miyada, Erica Regina Ribeiro Sady, Rosana Perim Costa, Leopoldo Piegas, Erlon Oliveira de Abreu-Silva, Alexandre Schaan de Quadros, Camila Weschenfelder, Júlia Lorenzon dos Santos, Gabriela Corrêa Souza, Suena Medeiros Parahiba, Ana Paula Trussardi Fayh, Danielle Soares Bezerra, Ana Paula Perillo Ferreira Carvalho, Malaine Morais Alves Machado, Sandra Mary Lima Vasconcelos, Jéssika Araújo, José Albuquerque de Figueiredo Neto, Luciana Pereira Pinto Dias, Francisca Eugenia Zaina Nagano, Cássia Cristina Paes de Almeida, Annie Seixas Bello Moreira, Débora Pinto Gapanowicz, Eduardo Purgatto, Marcelo Macedo Rogero, Geni Rodrigues Sampaio, Elizabeth Aparecida Ferraz da Silva Torres, Graziela Biude Silva Duarte, Alexandre Biasi Cavalcanti

**Affiliations:** 1 HCor Research Institute (IP-HCor), Hospital do Coração (HCor), Abílio Soares Street, 250, 12th floor, São Paulo, SP Zip Code 04004-050 Brazil; 2 Graduate Program in Health Sciences (Cardiology), Instituto de Cardiologia/Fundação Universitária de Cardiologia (IC/FUC), Porto Alegre, Rio Grande do Sul Brazil; 3 Health Knowledge Implementation Laboratory (LICS), Hospital do Coração (HCor), São Paulo, São Paulo Brazil; 4 Division of Cardiology, Hospital do Coração (HCor), São Paulo, São Paulo Brazil; 5 Hemodynamics Service, Hospital do Servidor Público Estadual (HSPE), São Paulo, São Paulo Brazil; 6 Division of Nutrition, Hospital de Clínicas de Porto Alegre (HCPA), Porto Alegre, Rio Grande do Sul Brazil; 7 Post-Graduation Program in Food, Nutrition and Health, School of Medicine, Universidade Federal do Rio Grande do Sul (UFRGS), Porto Alegre, Rio Grande do Sul Brazil; 8 Department of Nutrition, Universidade Federal do Rio Grande do Norte (UFRN), Natal, Rio Grande do Norte Brazil; 9 Faculty of Health Science of Trairi, Universidade Federal do Rio Grande do Norte (FACISA-UFRN), Santa Cruz, Rio Grande do Norte Brazil; 10 Clinical Nutrition Unit, Hospital de Clínicas, Universidade Federal de Goiás (HC-UFG/EBSERH), Goiânia, Goiás, Brazil; 11 Faculty of Nutrition, Universidade Federal de Alagoas (UFAL), Maceió, Alagoas Brazil; 12 Department of Cardiology, Universidade Federal do Maranhão (UFMA), São Luiz, Maranhão Brazil; 13 Complexo Hospital de Clínicas da Universidade Federal do Paraná (HC-UFPR), Curitiba, Paraná, Brazil; 14 Universidade Federal do Paraná (UFPR), Curitiba, Paraná, Brazil; 15 Instituto Nacional de Cardiologia (INC), Rio de Janeiro, Rio de Janeiro Brazil; 16 Department of Food Science and Experimental Nutrition/Food Research Center, Faculty of Pharmaceutical Sciences, Universidade de São Paulo (USP), São Paulo, São Paulo Brazil; 17 Department of Nutrition, School of Public Health, Universidade de São Paulo (USP), São Paulo, São Paulo Brazil; 18 Department of Food Science and Experimental Nutrition, Faculty of Pharmaceutical Science, Universidade de São Paulo (USP), São Paulo, São Paulo Brazil

**Keywords:** Myocardial infarction, Nuts, Atherosclerosis, Secondary prevention, Diet, Healthy, Arachis, Bertholletia, Anacardium

## Abstract

**Background:**

Nut consumption has been related to improvements on cardiometabolic parameters and reduction in the severity of atherosclerosis mainly in primary cardiovascular prevention. The objective of this trial is to evaluate the effects of the Brazilian Cardioprotective Diet (***DI****eta* ***CA****rdioprotetora* ***Br****asileira*, DICA Br) based on consumption of inexpensive locally accessible foods supplemented or not with mixed nuts on cardiometabolic features in patients with previous myocardial infarction (MI).

**Methods:**

DICA-NUTS study is a national, multicenter, randomized 16-week follow-up clinical trial. Patients over 40 years old with diagnosis of previous MI in the last 2 to 6 months will be recruited (*n* = 388). A standardized questionnaire will be applied to data collection and blood samples will be obtained. Patients will be allocated in two groups: Group 1: DICA Br supplemented with 30 g/day of mixed nuts (10 g of peanuts, 10 g of cashew, 10 g of Brazil nuts); and Group 2: only DICA Br. The primary outcome will consist of LDL cholesterol means (in mg/dL) after 16 weeks of intervention. Secondary outcomes will consist of other markers of lipid profile, glycemic profile, and anthropometric data.

**Discussion:**

It is expected that DICA Br supplemented with mixed nuts have superior beneficial effects on cardiometabolic parameters in patients after a MI, when compared to DICA Br.

**Trial registration:**

ClinicalTrials.gov Identifier NCT03728127. First register: November 1, 2018; Last update: June 16, 2021. World Health Organization Universal Trial Number (WHO-UTN): U1111-1259-8105.

**Supplementary Information:**

The online version contains supplementary material available at 10.1186/s13063-021-05494-0.

## Administrative information

The order of the items has been modified to group similar items (see http://www.equator-network.org/reporting-guidelines/spirit-2013-statement-defining-standard-protocol-items-for-clinical-trials/).

**Table:** 

Title {1}	Effects of a Brazilian Cardioprotective Diet and Nuts on Cardiometabolic Parameters after Myocardial Infarction: study protocol for a randomized controlled clinical trial.
Trial registration {2a and 2b}.	Brazilian Cardioprotective Diet and Nuts in Post-acute Myocardial Infarction (DICA-NUTS). Trial Registration: ClinicalTrials.gov Identifier NCT03728127. First register: November 1, 2018; Last update: June 16, 2021. World Health Organization Universal Trial Number (WHO-UTN): U1111-1259-8105
Protocol version {3}	Fourth version (approved by HCor-REC in 05/15/2019).
Funding {4}	Hospital do Coração (HCor); *Programa de Apoio ao Desenvolvimento Institucional do Sistema Único de Saúde* (PROADI-SUS); Brazilian Ministry of Health.
Author details {5a}	^1^ HCor Research Institute (IP-HCor), Hospital do Coração (HCor), São Paulo, São Paulo, Brazil. ^2^ Graduate Program in Health Sciences (Cardiology), Instituto de Cardiologia/Fundação Universitária de Cardiologia (IC/FUC), Porto Alegre, Rio Grande do Sul, Brazil. ^3^ Health Knowledge Implementation Laboratory (LICS), Hospital do Coração (HCor), São Paulo, São Paulo, Brazil. ^4^ Division of Cardiology, Hospital do Coração (HCor), São Paulo, São Paulo, Brazil. ^5^ Hemodynamics Service, Hospital do Servidor Público Estadual (HSPE), São Paulo, São Paulo, Brazil. ^6^ Division of Nutrition, Hospital de Clínicas de Porto Alegre (HCPA), Porto Alegre, Rio Grande do Sul, Brazil. ^7^ Post-Graduation Program in Food, Nutrition and Health, School of Medicine, Universidade Federal do Rio Grande do Sul (UFRGS), Porto Alegre, Rio Grande do Sul, Brazil. ^8^ Department of Nutrition, Universidade Federal do Rio Grande do Norte (UFRN), Natal, Rio Grande do Norte, Brazil. ^9^ Faculty of Health Science of Trairi, Universidade Federal do Rio Grande do Norte (FACISA-UFRN), Santa Cruz, Rio Grande do Norte, Brazil. ^10^ Clinical Nutrition Unit, Hospital de Clínicas, Universidade Federal de Goiás (HC-UFG/EBSERH), Goiânia, Goiás, Brazil. ^11^ Faculty of Nutrition, Universidade Federal de Alagoas (UFAL), Maceió, Alagoas, Brazil. ^12^ Department of Cardiology, Universidade Federal do Maranhão (UFMA), São Luiz, Maranhão, Brazil. ^13^ Complexo Hospital de Clínicas da Universidade Federal do Paraná (HC-UFPR), Curitiba, Paraná, Brazil. ^14^ Universidade Federal do Paraná (UFPR), Curitiba, Paraná, Brazil. ^15^ Instituto Nacional de Cardiologia (INC), Rio de Janeiro, Rio de Janeiro, Brazil. ^16^ Department of Food Science and Experimental Nutrition/Food Research Center, Faculty of Pharmaceutical Sciences, Universidade de São Paulo (USP), São Paulo, São Paulo, Brazil. ^17^ Department of Nutrition, School of Public Health, Universidade de São Paulo (USP), São Paulo, São Paulo, Brazil. ^18^ Department of Food Science and Experimental Nutrition, Faculty of Pharmaceutical Science, Universidade de São Paulo (USP), São Paulo, São Paulo, Brazil.
Name and contact information for the trial sponsor {5b}	Bernardete Weber Hospital do Coração (HCor) Abílio Soares Street, 250, 11th floor. São Paulo. SP. Brazil. Zip Code 04004-050 Phone: + 55(11) 3053 6611 – extension 8114 Email: bweber@hcor.com.br
Role of sponsor {5c}	The sponsor had approved the final version of this manuscript and the decision to submit the report for publication.

## Introduction

### Background and rationale {6a}

Ischemic heart disease (IHD) is the most common cause of mortality worldwide [1]. Myocardial infarction (MI) is the principal responsible for deaths from IHD [2], and it is expected that, within 1 year after a MI at ≥ 45 years of age, 18% of men and 23% of women will die in developed countries [1].

In addition to physiological features including high blood pressure, high blood cholesterol, and high blood glucose, dietary factors have been associated with a substantial number of deaths from heart diseases [3], and improving global eating patterns might prevent premature mortality from IHD [4]. Thus, foods and dietary patterns related to primary and secondary cardiovascular prevention are of great scientific interest.

Mediterranean diet is related to lower cardiovascular mortality [5], and the effects of its adaptations/variations on cardiometabolic features in post-MI individuals have been described [6–8]. However, despite regional adaptations, many Mediterranean diet foods are not widely available, may be expensive, or are not part of local eating habits in Brazil [9]. In this scenario, the Brazilian Cardioprotective Diet (***DI****eta* ***CA****rdioprotetora* ***Br****asileira*, DICA Br) was developed, based on a feasible dietary prescription guided by nutritional recommendations and on a ludic strategy for improving dietary compliance in adults with any atherosclerotic cardiovascular disease. In general, the DICA Br features nutritional recommendations that are feasible for the Brazilian population. Accordingly, the diet composition allows for the easy access and full use of foods, in addition to the prioritization of regional foods that are culturally accepted by the patients (rice, bean, soy oil, and Brazilian fruits and vegetables) [10–12].

Nuts and peanuts are rich in vegetal proteins, unsaturated fatty acids, and other bioactive components, such as dietary fibers, minerals, vitamins, phytosterols, and phenolic compounds [13, 14]. Furthermore, nut consumption has been related to lower IHD incidence [15]. Systematic reviews and metanalyses from randomized trials have shown the effects of different nuts and peanut intake on cardiometabolic features in primary cardiovascular prevention [16, 17] but few clinical trials have been conducted in individuals with previous IHD [8, 18–21]. In addition, these studies show discrepancies in the results, possibly due to different sample sizes and differences in the dietary pattern adopted for both intervention and control groups, intervention duration, design, population, and type of nuts evaluated [8, 16–19].

Guidelines for secondary cardiovascular prevention have suggested that 30 g/day of nut intake may be related to benefits for individuals already affected by a MI [22, 23]. However, references that support this recommendation were cited from observational or experimental studies conducted in primary prevention [24, 25]. DICA Br was effective in improving the diet quality of individuals in secondary cardiovascular prevention but did not change cardiometabolic risk factors [12]. Moreover, the intake of nuts is not genuinely stimulated in DICA Br, which does not emphasize the consumption of specific foods individually.

Considering that DICA Br is a regional and feasible dietary pattern, but it was assessed in a heterogeneous population, and considering also that benefits of consuming different nuts are not well established in the secondary cardiovascular prevention setting (mainly in post-MI), we designed a randomized clinical trial to evaluate and compare the effects of DICA Br supplemented or not with mixed nuts widely available in Brazil on cardiometabolic parameters in individuals with previous MI.

### Objectives {7}

Our primary objective is to evaluate the effect of two dietary approaches (DICA Br supplemented or not with 30 g/day of mixed nuts) on low-density lipoprotein cholesterol (LDL-c) levels in patients with previous MI after 16 weeks of follow-up.

The secondary objective is to evaluate the effects of both dietary approaches on other lipid profile variables, glycemic, and anthropometric features after 16 weeks of follow-up.

### Trial design {8}

The DICA-NUTS study is a national, multi-centered, randomized, open-label, parallel-group (1:1 allocation ratio), and superiority 16-week clinical trial with blinded outcome assessment.

## Methods: participants, interventions, and outcomes

### Study setting {9}

The study will be conducted in investigation sites across 4 Brazilian regions (Northeast, Southeast, South, and Midwest regions). HCor Research Institute (IP-HCor, São Paulo, Brazil) is responsible for the protocol and coordination of this trial. All investigation sites approached to take part in this study must count on a staff of registered dietitians to provide the dietary proposed intervention, as well as the physical structure/equipment to allow anthropometric and laboratory assessments.

The study population will include patients with previous diagnosis of MI identified in Hemodynamic Services, outpatient cardiology clinics, or during the period of hospitalization. Outpatients post-MI who volunteered for the trial may be also included. The procedures of randomization, allocation, and follow-up will take place at outpatient clinics of Nutrition at each investigation site.

### Eligibility criteria {10}

Inclusion criteria are as follows: male and female patients over 40 years old with diagnosis of previous MI in the last 2 to 6 months and who have signed an informed consent to participate. Diagnosis of both ST-Elevation MI (STEMI) and non-STEMI defined according to the guidelines [22, 23, 26, 27] must be confirmed at the baseline visit through medical records signed by a cardiologist.

Exclusion criteria are as follows: coronary artery bypass graft (CABG) surgery in the upcoming 16 weeks; acquired immunodeficiency syndrome; chronic inflammatory diseases and chronic use of anti-inflammatory, immunosuppressive, and/or anticonvulsant medications; active cancer (in any stage of current treatment); pregnancy or breastfeeding women; drug and alcohol abuse; physical disabilities that may impair anthropometric assessment; morbid obesity (body mass index [BMI] ≥ 40 kg/m^2^); history of allergy to nut intake; dietary use of nutritional supplements based on dietary fibers, polyunsaturated fatty acids (PUFA) omega-3, phytosterols, multivitamins, and probiotics; and participation in other clinical trials whose intervention may interfere with outcomes. Individuals who regularly eat nuts/oilseeds more than 3 times a week (evaluated by simply asking the consumption during the screening process) should not be included in the study.

To avoid dropout and attrition, we will provide investigator sites with strategies such as the possibility of rescheduling study visits within a 15-day range of both anticipation/delay in appointments, adaptation of financial compensation for transport to study visits according to costs (if that should be the impediment for participation), and motivational talks. If the participant chooses to end participation, investigators will still try to schedule the final study appointment (respecting the intention-to-treat nature of the protocol). When contact with participants is impaired throughout the study (ex. change of contact information), investigators will reach out to the person informed as point of contact during the baseline visit. Dropouts will be considered, hence, only when there is a lack of data in the last study visit, after all attempts of contact with patients are considered as failures to reach out to the patient.

### Who will take informed consent? {26a}

This study complies with national and international good clinical practices (Brazilian RDC 466/12, International Council for Harmonisation/Good Clinical Practices [ICH/GCP], World Health Organization [WHO], and Declaration of Americas). The investigator who approaches eligible participants to the study is responsible for obtaining the written informed consent from all participants (Supplementary Material, Appendix II).

We will financially compensate participants for transportation costs throughout the study, as well as provide them with meals following blood testing in the first and last visit for data collection.

### Additional consent provisions for collection and use of participant data and biological specimens {26b}

This is not applicable. We will not apply additional consent provisions for collection. If this trial subsidizes any further analysis not described in this primary plan, researchers will need to re-get consent from participants.

### Interventions

#### Explanation for the choice of comparators {6b}

DICA Br is a dietary prescription originally designed for secondary cardiovascular prevention guided by nutritional content recommendations as per guidelines and composed by locally available foods in each Brazilian region. Trial participants will be assigned to either of two groups, intervention (Group 1) or control/comparator (Group 2), both provided with dietary counseling based on the recommendations of the DICA Br—which is part of the Brazilian Cardioprotective Nutritional (BALANCE) Program [10–12].

As a strategy to facilitate adherence to the DICA Br, the registered nutritionist counseling prioritizes local and affordable foods and uses heart symbols of different colors a ludic approach to help raise awareness of healthy proportions between food groups [10–12]. The colors (green, yellow, and blue) are those of the Brazilian flag. In summary, foods in the green group are rich in vitamins, minerals, and dietary fibers and have a low energetic density, saturated fatty acids (SFA), and dietary sodium content; the yellow group is mainly composed by foods rich in carbohydrates and vegetal fats; and the blue group comprises animal sources of proteins, with a higher content of dietary sodium, cholesterol, SFA, and energetic density. A fourth group colored red is composed by ultra-processed foods. In allusion to the absence of the color red in the Brazilian flag, foods from this group should be avoided as often as possible.

The registered nutritionists will plan their counseling based on the premises described as follows (Table 1):

**Table 1:** Rationale for dietary counseling

Milestones	Registered dietitian activities	
Baseline visit	Daily energy requirements	Pocket formula [10–12] according to the participants’ BMI classification at baseline visit: • 20 kcal/kg for overweight and obese participants (BMI ≥ 25 kg/m^2^, targeting weight loss); • 25 kcal/kg for normal BMI participants (BMI 18.5 to 24.9 kg/m^2^, targeting weight maintenance); • 32 kcal/kg for those with low BMI (BMI < 18.5 kg/m^2^, targeting weight gain).
Baseline visit	Semi-quantitative nutritional counseling	• Dietary counseling to raise awareness of the colored food groups: encourage participants to increase intake of foods in the green group, moderate foods in the yellow group and reduce intake of foods in the blue group in the next 30 days; • The intake of ultra-processed foods (red group) will be strongly discouraged throughout the trial; • Participants will receive an educational material with a brochure to aid food choices (according to color groups), and a recipe book (with tested recipes, adapted to the protocol).
Visits 2, 3, and 4	Quantitative nutritional counseling	• Distribution of daily portions of each food group (colors green, yellow, and blue) into the participants’ routine according to the energy requirements planned at baseline visit; • Use of pre-elaborated menus that balance the amount of green, yellow, and blue food servings (1400 to 2400 kcal) [11] as examples for counseling; • Encourage patients to make their own combinations using the educational materials supplied, so to achieve the energetic goal established at baseline visit;
Visit 5	Qualitative nutritional counseling	• Make final adjustments to counseling and encourage participants to maintain the new healthy patterns acquired after the study’s finished.

*BMI* body mass index

Table 2 shows the lowest caloric menu available (1400 kcal/day) as an example of DICA Br dietary prescription, including dietary composition and the amount of food servings according to color groups [10–12]. Supplementary material shows food group (portions/day) standard harmonization according to energy requirement ranges for DICA Br prescription (Table S1) and an example of a menu/daily food distribution and corresponding number of colored food groups, as a ludic strategy for improving compliance (Chart S2).

**Table 2:** Example of dietary prescription (DICA Br and DICA Br - NUTS) including dietary composition and food servings according to DICA Br color groups

	DICA Br composition	DICA Br - NUTS
Total energy, in kcal/day	1400	1600
Carbohydrate, in % TE	57	50
Protein, in % TE	23	23
Total fat, in % TE	20	27
Saturated fatty acids, in % TE	7	9
Monounsaturated fatty acids, in % TE	5	10
Polyunsaturated fatty acids, in % TE	5	7
Dietary cholesterol (mg)	93	94
Dietary fiber (g)	20	22
Green group portions	9	9
Yellow group portions	6	7
Blue group portions	2	2

*% TE* percentage of total energy, *DICA Br DIeta CArdioproterora Brasileira*, *DICA Br - NUTS* DICA Br + 30 g of mixed nuts (10 g of peanuts, 10 g of cashew, and 10 g of Brazil nut)

#### Intervention description {11a}

Group 1 (DICA Br plus 30 g of mixed nuts) participants will be advised to supplement their diet with a daily amount of 30 g of mixed nuts over the intervention period. The research team will provide the participant with individual packages containing a mix of 10 g of toasted cashew nuts, 10 g of raw and peeled peanuts, and 10 g of raw Brazil nuts (all without salt). Each investigator center will receive precision scales with 0.1 to 2000 g accuracy to reassure this procedure. Participants will be advised to eat the nuts plain as a first-line counseling but will be free to use them as culinary ingredients, in case of low adherence (i.e., together with yogurt, mixed with rice or other cereals, etc.), as a secondary line. We will train care providers to counsel such modifications to diet. Participants will be also counseled about adequate storage of the nuts provided.

All nuts will be acquired from local and regional farmers (peanuts: state of Rio Grande do Sul, Southern region of Brazil; cashew nuts: state of Rio Grande do Norte, Northeast region of Brazil; and Brazil nuts: state of Mato Grosso do Sul, Midwestern region of Brazil). Centesimal composition, fatty acid profile, and minerals’ content of the nuts provided in the study, including the methodology used for chemical analysis are described in Supplementary Materials (Table S2 and Appendix I).

Group 2 (only DICA Br) will be advised to follow the recommendations of the DICA Br, and to avoid eating nuts and oilseeds of any type over the intervention period.

Diets will not be isocaloric between intervention and controls, given that while Group 2 will be prescribed a diet according to the energetic goals established only (ex. 1400 kcal/day), Group 1 will be prescribed the same and then added the mixed nuts (ex. 1400 kcal/day + mixed nuts) (Table 2).

All participants will be instructed to maintain and not modify their physical activity levels during the study.

#### Criteria for discontinuing or modifying allocated interventions {11b}

This is an intention-to-treat trial, so we will not exclude data from the database in case of intervention discontinuation and will remain following the participants even without any intervention for the remaining study duration, unless the participant demand not to be followed. We expect a few possible reasons for intervention discontinuation, such as upon request and/or low adherence to intervention; presence of adverse events; presence of any factor that may require specific nutritional therapy other than the provided by the trial; or when the participant develops any of the exclusion criteria. Patients will be prompted to complete the follow-up. Participants who discontinue intervention will be asked to come for the final assessment, for blood sample and data collection.

In case the investigator cannot locate the participant after being randomized, we will train the investigation site to try contact several times. If after exhaustive efforts and the participant’s outcome cannot be determined, we will consider the data as missing (not excluded from the database). Participants may be reallocated among investigation sites when needed (i.e. if a participant moves from one city to another), although data must be analyzed within original randomization site.

#### Strategies to improve adherence to interventions {11c}

Investigators will monitor adherence to intervention through the return of the individual mixed nuts packages (Group 1) and through 24-h food records (both groups) provided at each follow-up visit. In addition, investigators will contact all participants for remembering the visits scheduled and, if necessary, will re-scheduled it according to the participant’s preference. In these calls, the importance of interventions’ adherence will be reinforced.

#### Relevant concomitant care permitted or prohibited during the trial {11d}

The registered nutritionists will encourage participants to avoid in their routines (or to eat them as little as possible) any other amount of nuts and oilseeds other than the mix provided in the study.

#### Ancillary and provisions for post-trial care {30}

This is not applicable. No specific provisions for post-trial care were considered in this trial, due to the characteristic of the intervention, the follow-up period and the outcomes evaluated. All harms and adverse events (AE) will be dealt properly, and if the participants need any health care due to participation in the study, they will be referred to the respective referral services. If the superiority of DICA Br supplemented with mixed nuts is confirmed, participants in the control group will be advised to include these foods in their routine.

#### Outcomes {12}

##### Primary outcome

The primary outcome will consist of LDL-c means (mg/dL) at 16 weeks.

##### Secondary outcomes

- Means of other measures of lipid profile (total cholesterol [TC], high-density lipoprotein cholesterol [HDL-c], very low-density lipoprotein cholesterol [VLDL-c], non-HDL cholesterol [NHDL-c], fasting triglycerides [TG], Castelli indexes I and II, and TG/HDL-c ratio) at 16 weeks;
- Means of glycemic profile variables (fasting glucose [FG], fasting insulin [FI], glycated hemoglobin [HbA1c], and Homeostases Model Assessment-Insulin Resistance [HOMA-IR]) at 16 weeks;
- Means of anthropometric measures (body weight, BMI, waist circumference, hip circumference, waist to hip ratio, and waist to height ratio) at 16 weeks.

#### Participant timeline {13}

Patients will be invited personally or by telephone to participate in the study. For the individuals who meet the inclusion criteria, a visit will be scheduled with the research team for eligibility confirmation. All individuals will be advised to fast overnight for 12 h before the baseline visit.

After eligibility confirmation and informed consent process, patients will undergo a baseline assessment and a blood sample will be collected. The baseline assessment comprises questionnaires on sociodemographic and lifestyle data (smoking, alcohol intake, and level of physical activity), medical history (including previous diagnosis and drug prescriptions), and dietary patterns. We will also assess anthropometric data (body weight, body height, waist and hip circumferences).

After randomization and once allocation has been completed, the patient will be appropriately advised according to the intervention designated: control diet (DICA Br) or intervention diet (mixed nuts added to DICA Br). Patients will be followed for a period of 4 months (16 weeks), and follow-up visits will be scheduled at 30 days, 60 days, 90 days, and 120 days (final visit), when a new blood sample will be collected. Table 3 summarizes the proposed intervention and study flow.

**Table 3:**
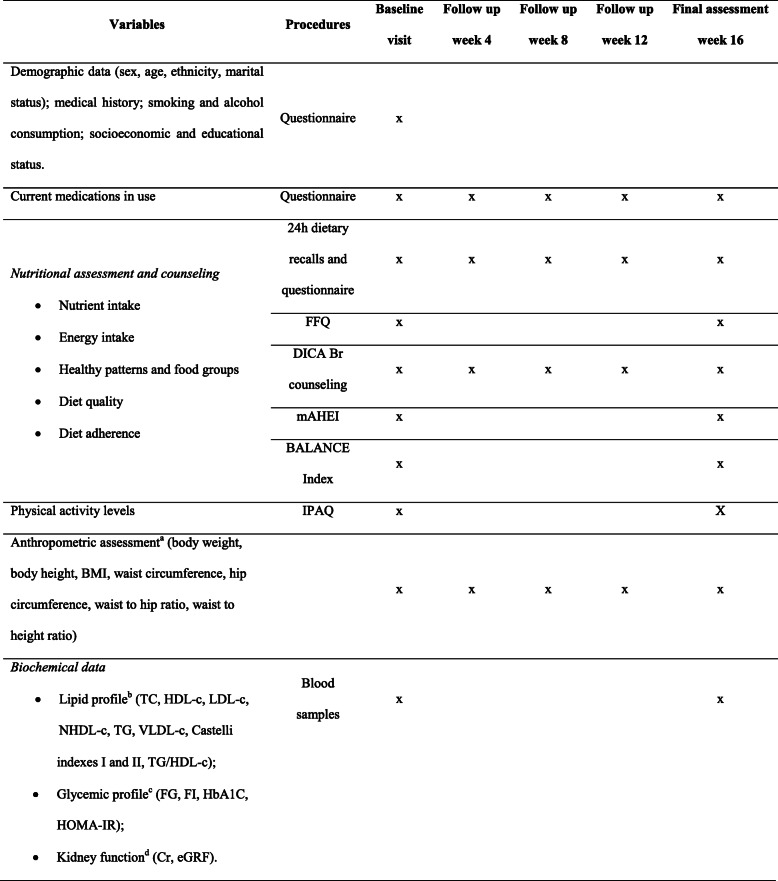
Study flowchart and summary of data collection

Each investigation site will be responsible for data collection and transcription into both paper and electronic case report form (CRF), at the study website.

#### Sample size {14}

Based on the BALANCE study, the observed correlation between baseline LDL-c and final LDL-c (16 weeks) was 0.6, and the standard deviation was 24. A sample size estimated at 352 participants would guarantee a power of 80% to detect a minimum difference of 6 mg/dL in LDL-c between groups, with two-tailed alpha of 5%. Adding 10% considering possible dropouts, the final sample size will consist of 388 individuals.

#### Recruitment {15}

Each investigator center will be responsible for the promotion of the study in the community and for participant recruitment according to eligibility criteria and to regional aspects of population. Investigators may promote the study via social media/TV or other media available, via partnerships with community healthcare services or within the institution’s Hemodynamic Services, Cardiology inpatient and outpatient services. Voluntary patients will also be accepted in the study. In all cases, we will ask investigator sites to fill in a screening log of each approach, including reasons for declining participation, so it will be possible to re-evaluate recruitment strategies throughout the study whenever there is difficulty in achieving recruitment targets.

## Assignment of interventions: allocation

### Sequence generation {16a}

The coordinator center will generate the allocation sequence of 1:1 via validated software with random permuted blocks and stratification, according to the investigator center, to reduce the predictability of a random sequence. Only the study coordination will have access to randomization sequence generation.

### Concealment mechanism {16b}

The allocation concealment mechanism will be central, via website (http://dicanuts.hcor.novatela.com.br).

### Implementation {16c}

The web-based automated randomization system, available 24 h a day, was developed by the IP-HCor. Investigators will need to access the study website, then fill in the electronic CRF and confirm eligibility criteria to be granted access to randomization.

## Assignment of interventions: blinding

### Who will be blinded {17a}

Because of the dietary intervention proposed, this will be an open trial and both participants and care providers will have awareness of dietary approach assigned to each group. Researchers and statisticians will be blinded to the intervention groups during statistical analysis, as well as staff involved with biochemical sample analysis.

### Procedure for unblinding if needed {17b}

This is not applicable. This will be an open-label randomized trial.

## Data collection and management

### Plans for assessment and collection of outcomes {18a}

We will train all researchers (in person or remotely) according to the standard operating manual created for this trial at each investigation site, with special focus on variables that may suffer deviations from inter- and intra-observer variations, such as anthropometric and food consumption measurements.

#### Demographic data

A standardized questionnaire will be administered to all participants for the collection of demographic variables (age, sex, ethnicity, marital status). Socioeconomic and education data will be evaluated according to the Brazilian Criteria for Economics Classification [28].

#### Clinical and lifestyle data

- Medical history: Data on type of MI (STEMI or non-STEMI), family history of premature IHD, approximate date of the MI, time of MI diagnosis, history, and approximate date of previous outcomes (i.e., MI before 180 days, stroke, angioplasty with or without stenting, previous CABG) will be collected . Dyslipidemia, hypertension, and diabetes mellitus types 1 and 2 will be registered according to previous reports of medical diagnosis and/or the use of medications to treat each of these conditions.
- Medications: Data about current drugs in use will be collected. Specifically, regarding use of statins and other lowering lipid drugs, we will ask for type, dosage, and frequency of use.
- Smoking and alcohol intake: Smoking will be defined as a categorical variable (never, current or past smoking) [29];
- Excessive alcohol intake will be notified according to sex [30]:
  - Men: intake of ≥ 30 g of ethanol per day; or ≥ 5 doses in approximately 2 h; or ≥ 5 doses a day of alcoholic beverages in the last month;
  - Women: intake of ≥ 15 g of ethanol per day; or ≥ 4 doses of alcoholic beverages in approximately 2 h; ≥ 5 doses a day of alcoholic beverages in the last month.
- Physical activity: We will define levels of physical activity as categorical variables according to the International Physical Activity Questionnaire (IPAQ) long version translated and validated into the Portuguese language [31].

#### Anthropometry

Body weight (in kg) should be obtained with participants’ barefoot and wearing minimal clothing. Body height (in cm) should be obtained with participants barefoot in the standing position, and with both arms hanging freely at the side with palms facing thighs. BMI will be calculated, and the nutritional status will be defined according to WHO [32]. Waist circumference and hip circumference (both in cm) should be assessed with a tape measure of resistant, inelastic, and flexible material, with an accuracy of 0.1 cm. Waist circumference will be obtained through the midpoint between the lower edge of the costal arch and the iliac crest in the middle axillary line; the hip circumference will be defined as the measurement with the highest value in the hip region, with the patient’s legs joined. Waist to hip ratio and waist to height ratio will be calculated; abdominal obesity will be classified according to WHO [33] and Ashwell [34] criteria.

#### Biochemical data

Blood samples will be collected, and biochemical assessment will be carried out according to standardized techniques by the clinical analysis laboratories referenced for each center site. TC (mg/dL), HDL-c (mg/dL), TG (mg/dL), FG (mg/dL), FI (mU/L), HbA1c (%), and serum creatinine (Cr, mg/dL) will be obtained directly from blood samples. LDL-c (in mg/dL) will be obtained from Martin’s mathematical formula [35]. VLDL-c (mg/dL), NHDL-c (md/dL), TG/HDL-c ratio (mg/dL), TC/HDL-c ratio (Castelli I index, mg/dL), LDL-c/HDL-c ratio (Castelli II index, mg/dL) and HOMA-IR will be defined according to specific mathematical formulas. Estimated glomerular filtration rate (eGFR, mL/min/1.73 m^2^) will be obtained by mathematical equations specifically for men and women according to the Modification of Diet in Renal Disease Study [36].

#### Dietary assessment

We will use a 24-h dietary recall and a food frequency questionnaire (FFQ) to record data on nutrient intake and eating patterns [37], and both will be applied by trained nutritionists. All data will be recorded in a specific software site (*Sistema Vivanda de Alimentação*®, São Paulo, Brazil) [38]. The same photo album containing images of standardized food portion sizes used in BALANCE Program [11] will be used to assist food intake evaluation.

Diet quality will be evaluated according to the modified Alternative Healthy Eating Index (mAHEI) [39], previously used in the BALANCE trial [12]. Adherence to the dietary prescription will be evaluated according to the BALANCE Index, validated specifically for DICA Br assessment [40].

Each investigation site will be responsible for data collection and transcription into both paper and electronic CRF, at the study website. Variables, procedures, and milestones for data collection are summarized in Table 4.

**Table 4:** Data collection plan

Variables	Procedures	Baseline visit	Follow-up week 4	Follow-up week 8	Follow-up week 12	Final assessment week 16
Demographic data (sex, age, ethnicity, marital status); medical history; smoking and alcohol consumption; socioeconomic and educational status.	Questionnaire	x				
Current medications in use	Questionnaire	x	x	x	x	x
*Nutritional assessment and counseling* • Nutrient intake • Energy intake • Healthy patterns and food groups • Diet quality • Diet adherence	24-h dietary recalls and questionnaire	x	x	x	x	x
	FFQ	x				x
	DICA Br counseling	x	x	x	x	x
	mAHEI	x				x
	BALANCE Index	x				x
Physical activity levels	IPAQ	x				X
Anthropometric assessment^a^ (body weight, body height, BMI, waist circumference, hip circumference, waist to hip ratio, waist to height ratio)		x	x	x	x	x
*Biochemical data* • Lipid profile^b^ (TC, HDL-c, LDL-c, NHDL-c, TG, VLDL-c, Castelli indexes I and II, TG/HDL-c); • Glycemic profile^c^ (FG, FI, HbA1c, HOMA-IR); • Kidney function^d^ (Cr, eGRF).	Blood samples	x				x

*FFQ* food frequency questionnaire, *DICA Br DIeta CArdioprotetora Brasileira*, *mAHEI* modified Alternative Healthy Eating Index, *BALANCE* Brazilian Cardioprotective Nutritional Program, *IPAQ* International Physical Activity Questionnaire, *BMI* body mass index, *TC* total cholesterol, *HDL-c* high-density lipoprotein cholesterol, *LDL-c* low-density lipoprotein cholesterol, *NHDL-c* non-HDL cholesterol, *TG* fasting triglycerides, *VLDL-c* very low-density lipoprotein cholesterol, *TG/HDL-c* ratio between TG and HDL-c, *FG* fasting glucose, *FI* fasting insulin, *HbA1c* glycated hemoglobin, *HOMA-IR* homeostasis model assessment-insulin resistance, *Cr* serum creatinine, *eGRF* estimated glomerular filtration rate

^a^General obesity defined as BMI ≥ 30 kg/m^2^ [32]; abdominal obesity defined as waist circumference ≥ 94 cm (men) and ≥ 80 cm (women), waist to hip ratio > 0.90 (men) and > 0.85 (women) [33], and/or waist to height ratio > 0.5 (both sexes) [34]

^b^VLDL-c calculated via formula TG/5; LDL-c calculated via Martin’s formula (LDL-c = TC-HDL-c –TG/X, where X ranges from 3.1 to 11.9 considering TG ≥ 400 mg/dl) [35]; NHDL-c calculated via formula TC – HDL-c; Castelli 1 index calculated via formula TC/HDL-c; Castelli II index calculated via formula LDL-c/HDL-c; TG/HDL-c ratio calculated by dividing TG for HDL-c

^c^HOMA-IR calculated via formula FG (mmol) × FI (UI/mL) ÷ 22.5

^d^eGRF (in mL/min/1.73 m^2^) calculated via formula GRF = 175 × [Cr^−1.154^] × [age^−0.203^] × [0.742, if women] × [1.210, if afro descendant]) [36]

### Plans to promote participant retention and complete follow-up {18b}

To avoid attrition and maintain stimuli, investigators will be trained to maintain close contact with participants between study appointments, and to remotely collect clinical and dietary data when participants cannot attend the scheduled assessments. Regarding biochemical and anthropometric variables, the participants will be asked about the possibility of having short study visits at their home (following all safety protocols) only for anthropometric measures and blood collection.

### Data management {19}

The objective of our clinical data management plan is to provide high-quality data by adopting standardized procedures to minimize the number of errors and missing data, and consequently, to generate an accurate database for analysis.

Data collection will be performed using electronic CRF via internet at the HCor Data Management System. The system has the following functions: patient registration, 24-h randomization with allocation concealment, data input, data cleaning, and data export for statistical analysis. Data are entered directly into the system by each center. All forms are electronically signed by the principal investigator of each center or by other appointed persons. Instructions for using the system will be made available to investigators.

Several strategies will be performed to generate completeness and correctness of the clinical data. Investigators attended a training session before the start of the study to standardize procedures, including data collection. Study support material will be available at all sites, and the investigators may contact the study coordinating center to solve issues or problems that may arise. Several problems can be detected by the system at the time of data entry. Subsequently, data monitoring will be performed by a data management team in the central office that looks for missing data and inconsistencies using routines implemented in R software.

Monthly, reports of recruitment will be presented for all screened patients by site: the number of days recruiting, number of patients screened and recruited per day, number of screened but not recruited patients, and the reason for non-recruitment. Follow-up data will be assessed, and missing, inconsistent, illogical, out of range, and discrepant data will be marked. Specifically, regarding dietary features, we will screen implausible energy intake according to the criteria: < 500 and > 4000 kcal/day. In addition, 10% of the food recalls will be evaluated in their entirety to confirm all information entered in the electronic CRF and to assess the need for further training. We will notify the investigation sites for corrections or justifications. Resolution of queries by the investigator will be updated in the database. If the investigator cannot provide a resolution, the reasons will be collected in a spreadsheet. The data management team is also responsible for helping to detect cases of protocol deviation. If such situations occur, we will program new training sessions at the site to revise the protocol.

The database will be locked as soon as all data are entered, and all discrepant or missing data are resolved in the database or if all efforts are employed and we consider that the remaining issues cannot be fixed. At this step, our statisticians will review the data before database locking. We will fill out a database lock checklist before locking the database to ensure the completion of activities. After that, the study database will be locked and exported for statistical analysis. At this stage, permission to access the study database will be removed and the database will be archived.

### Confidentiality {27}

We will guarantee confidentiality of data through codified identification of participants (ID) generated by randomization processes, name initials, and birth date. Personal information will not be disseminated and documents containing such information will be confidentially stored and assessed, according to GCP. We will only make the final trial dataset accessible to the allowed investigators.

### Plans for collection, laboratory evaluation, and storage of biological specimens for genetic or molecular analysis in this trial/future use {33}

Storage of blood samples are previewed for this protocol, in preparation for future studies (Supplementary Material, Appendix III).

## Statistical methods

### Statistical methods for primary and secondary outcomes {20a}

All analyses will follow the intention-to-treat principle. Continuous variables will be presented as means and standard deviations or medians and interquartile range. Categorical variables will be presented as relative and absolute frequencies. The primary outcome will be establishing the difference in LDL-c at 16 weeks after baseline visit between the DICA Br + 30 g of mixed nuts group and the DICA Br. The difference will be assessed with analysis of covariance (ANCOVA) model with LDL-c (16 weeks) as the outcome and treatment group, sites, and baseline LDL-c as covariates. We will also use the ANCOVA on the lipid profile (TC, HDL-c, VLDL-c, NHDL-c, TG, Castelli indexes I and II, and TG/HDL-c ratio) and glycemic profile (FG, FI, HbA1C, and HOMA-IR). Anthropometric measurements that will be collected at baseline, 4, 8, 12, and 16 weeks will be analyzed over the time by generalized estimating equations (GEE) according to the data distribution. For all effect parameters, 95% confidence intervals will be reported. All analyses will consider a two-tailed alpha of 5% and will be performed on the R statistical software.

### Interim analyses {21b}

This is not applicable. We will not perform interim analysis in this trial.

### Methods for additional analyses (e.g., subgroup analyses) {20b}

Pre-specified subgroup analyses will be conducted according to type of MI and previous comorbidities. Dietary variables will be adjusted for total energy intake according to the residual method [41]. Possible differences in nutrient intake, group foods, or dietary patterns according to study groups will be considered in adjusted analysis on the primary outcome.

### Methods in analysis to handle protocol non-adherence and any statistical methods to handle missing data {20c}

A multiple imputation approach will be applied as a method to handle missing data. Mixed model of repeated measures and ANCOVA without imputation will be conducted as sensitivity analyzes.

### Plans to give access to the full protocol, participant-level data, and statistical code {31c}

Granting public access to database will follow the data sharing policy of IP-HCor. Interested parties should write to the corresponding author, and database should be shared via email in a non-identifiable form, after data lock and main paper publications.

## Oversight and monitoring

### Composition of the coordinating center and trial steering committee {5d}

The trial steering committee will be composed by physicians and registered dietitian researchers. The coordinator center will be composed by physicians, registered dietitians, statisticians, a center site coordinator, a qualified data manager, and regulatory department professionals.

As main responsibilities, the coordinator center will generate the allocation sequence, perform site and data management and analysis, while investigation sites will enroll participants and assign them to interventions and follow-up. The principal investigator at each center leads and/or supervises the daily operation of the project at his/her participating center and may appoint a co-investigator and research coordinator. Most tasks can be delegated by the principal investigator to research professionals at the investigation center provided that the professionals are qualified for such tasks and that the delegation is clearly recorded with the name of the professional and their role. However, the principal investigator will be legally responsible for the study and is responsible for ensuring that the data will be properly collected and entered into the Study Data Management System.

### Composition of the data monitoring committee, its role and reporting structure {21a}

This is not applicable. Given the short duration and knowledge of minimal risks of intervention, there will be no formal data monitoring committee in this trial.

### Adverse event reporting and harms {22}

We will train all investigation sites to assess, manage, and report possible AE in every follow-up visit. We will classify AE as serious adverse events (SAE) or suspected unexpected serious adverse reaction (SUSAR) according to severity levels and report them to the Ethics Committee, following the ICH/GCP and national guidelines. If any of such events occur, researchers will promptly follow the participants until complete recovery and adjust/stop the intervention as required. We will also train investigation sites to monitor and report unanticipated problems such as equipment/document robbery at investigation sites, loss or robbery of nuts packages, or system general failures.

#### Expected adverse events

We expect the following probably minor AE in this protocol: food allergy reactions to the consumption of nuts; gastrointestinal symptoms (nausea and vomiting, flatulence, diarrhea, abdominal pain or congestion, etc.); choking and/or suffocation events; and food poisoning events/nutrient toxicity. We will manage such event as described as follows (Table 5):

**Table 5:** Strategies for adverse events management according to the expected event

Expected event	Strategies for adverse events management
Food allergy reactions to the consumption of nuts (Group 1)	Known allergy to nuts is part of exclusion criteria in this trial. If cases of unknown allergy are discovered, we will ask participants to immediately stop intervention and provide proper treatment until complete cessation of symptoms.
Gastrointestinal symptoms	The registered nutritionist will be trained to adjust dietary counseling according to the symptoms reported.
Choking and/or suffocation events (both groups)	We will train researchers to assess oral motor abilities of participants to chew and swallow foods during the screening process, so to diminish the probability of choking and/or suffocation events. To those who experience such conditions during the trial period, it will be possible to chance culinary preparations as to facilitate intake, as mentioned previously as a second line of counseling.
Food poisoning (both groups)	Since this is a dietary intervention-based trial, there is a chance of food poisoning in the study. Hygienic food preparation procedures will be stimulated by the care providers throughout the intervention period. To reduce the risk of poisoning from mycotoxins usually found in raw peanuts (Group 1), we will ask farmers to provide a report to ensure mycotoxin safety levels before every purchase.
Events related to blood testing or to the participants’ baseline cardiovascular condition	Bruises or phlebitis or new episodes of myocardial infarction are also expected. In presence of new MI, it may be necessary to stop intervention during the hospitalization and recovery period.

### Frequency and plans for auditing trial conduct {23}

This is not applicable. No procedures for auditing trial conduct are planned. However, the sponsor may require any information and reports during the trial conduction and after its finalization.

### Plans for communicating important protocol amendments to relevant parties (e.g., trial participants, ethical committees) {25}

The coordination team will communicate any protocol modifications to the investigation sites through monthly newsletters, periodic training, and online frequent communication. The Research Ethics Committee/Institutional Review Board (REC/IRB) will be also notified whenever needed.

## Dissemination plans {31a}

We will disseminate the results to all participants and involved care providers via general report after publication of results.

## Discussion

Nut and peanut consumption have been related to improvements on lipid profile [16, 42], glycemic control [43, 44], and weight loss/no weight gain [45, 46] in primary cardiovascular prevention. In addition, including these foods on daily menu have been associated with higher diet quality in primary [47] and secondary cardiovascular prevention [18]. It is expected that low-quality diets may account for more than 18% of all type 2 diabetes mellitus, stroke, and IHD costs in development countries, being the largest annual costs per capita attributed to low consumption of nuts and seeds [48]. Thus, improving dietary patterns at the expense of low-cost foods might reflect not only in individuals’ health, but also in the economic burden of the health care systems. Noteworthy is the fact that in DICA-NUTS trial all nuts provided will be acquired from local farmer’s cooperatives and small producers; if effective, the study may contribute also to local economic growth by stimulating the consumption of foods locally cultivated.

Nut consumption has been related to reducing the severity of atherosclerosis [49–51] by acting on intermediary mechanisms such as improving endothelial function, lipid metabolism, and glycemic markers, protecting both DNA and LDL-c from oxidation, decreasing inflammation, altering microbiota, and controlling body weight [52–54]. Nuts and seeds may improve serum lipids by reducing cholesterol absorption, increasing bile acid production by stimulation of 7-hydroxylase, inhibiting HMG-CoA reductase, and reducing postprandial lipidemia by their poor lipid bioaccessibility [53, 55]—which is also implicated on body weight control [51]. We chose LDL-c levels as our primary outcome due to its clinical relevance in the cardiovascular prevention setting, given that for each 1 mmol/L in LDL-c reduction, for example, it is expected a reduction of 20% in mortality rates due to coronary heart disease and 27% lower risk for non-fatal MI [56].

Lipid-lowering drugs are most beneficial when prescribed as adjunctive therapy with healthy diets [22, 57]; in this sense, nuts and their metabolites seem to potentialize the lipid-lowering effects of statins [21, 57–59]. It is expected that both intervention and control groups be balanced regarding lipid-lowering medication and other drugs considering the randomization process; however, it must be considered a possible role of medications in improving metabolic parameters in both groups, as the population evaluated are likely to be newly commenced on lipid-lowering medication.

Considering that each kind of nut presents a unique nutritional composition and taking into account that some clinical trials that evaluated only one type of nut showed negative results on metabolic features [18, 21, 60], the consumption of a daily mixed nuts seems to be more interesting. In addition, a mix could improve adhesion for not repeat the same food daily (considering a standardized portion of ~ 28 g, which may not be widely acceptable for who is not used to eat nuts daily) and could make the diet cheaper.

To conduct a dietary intervention-based trial is not without its challenges. The success of the trial depends on a strong adherence to the intervention, and such studies often have problems with adherence. Downer et al [61] mention a few predictors of higher short-term adherence to dietary interventions, such as female sex, older age, a non-diabetic and non-depressive status, normal weight, higher physical activity levels, not smoking, white ethnicity, higher socioeconomic status, and being married. When considering long-term adherence to diet, they found that a higher number of cardiovascular risk factors, larger waist circumference, lower physical activity levels, and lower total energy intake also predicted poorer adherence. In our trial, participants must have already presented a cardiovascular event and are more likely to present comorbidities and overweight, which leads to the anticipation of low adherence to the intervention in both groups (having to eat daily portions of nuts in Group 1, and not being able to eat ordinary foods which contain nuts in Group 2). For example, participants in the intervention group may forget to eat the daily portion of nuts or may get bored with the new dietary routine. Because of that, researchers in all investigation sites will need to monitor participants who show signs of low adherence over the follow-up visits, as well as those with a known risk of low adherence to change and intensify stimuli. We can use different strategies, such as counseling the participants in the intervention group to eat the daily portion of nuts always in the same period of the day (preferably linked to an everyday habit, such as medications or routine meals) and setting up alarms. We foresee additional team efforts to promote adherence to our protocol.

In conclusion, the randomized controlled DICA-NUTS trial was designed to evaluate the effects of a locally affordable diet based on ludic strategies and nutritional guidelines supplemented or not with mixed nuts in a sample of patients with previous MI. Although many studies have shown the beneficial effects of nuts and peanuts on cardiometabolic parameters in primary cardiovascular prevention, clinical trials on established IHD are scarce. Considering the relevance of DICA Br as a locally available cardioprotective dietary pattern [62], promoting improvements on it through the advice of including specific foods may contribute to the clinical management of patients at very high risk for a new cardiovascular event.

## Trial status

The DICA-NUTS trial is ongoing and includes 09 center sites in Brazil. Enrollment began in January 2019. As of June 2021, a total of 357 patients had been included in the study. Inclusions and follow-up of all participants are planned to continue until December 2021.

## Supplementary Information

**Additional file 1.**

## Abbreviations

% TE: Percentage of total energy
AE: Adverse events
BALANCE: Brazilian Cardioprotective Nutritional Program
BMI: Body mass index
CABG: Coronary artery bypass graft
CRF: Case report form
DICA Br: DIeta CArdioprotetora Brasileira (Brazilian Cardioprotective Diet)
eGFR: Estimated glomerular filtration rate
FG: Fasting glucose
FI: Fasting insulin
GEE: Generalized estimating equations
HbA1c: Glycated hemoglobin
HDL-c: High-density lipoprotein cholesterol
HOMA-IR: Homeostases Model Assessment-Insulin Resistance
ICH/GCP: International Council for Harmonisation/Good Clinical Practices
IHD: Ischemic heart disease
IPAQ: International Physical Activity Questionnaire
IP-HCor: HCor Research Institute
LDL-c: Low-density lipoprotein cholesterol
MI: Myocardial infarction
NCEP: National Cholesterol Education Program
NHDL-c: Non-HDL cholesterol
PUFA: Polyunsaturated fatty acids
REC/IRB: Research Ethics Committee/Institutional Review Board
SAE: Serious adverse events
SFA: Saturated fatty acids
STEMI: ST-Elevation Myocardial Infarction
SUSAR: Suspected unexpected serious adverse reaction
TC: Total cholesterol
TG: Fasting triglycerides
VLDL-c: Very low-density lipoprotein cholesterol
WHO: World Health Organization
