# Correction to: Effects of a Brazilian cardioprotective diet and nuts on cardiometabolic parameters after myocardial infarction: study protocol for a randomized controlled clinical trial

**DOI:** 10.1186/s13063-021-05611-z

**Published:** 2021-09-29

**Authors:** Aline Marcadenti, Bernardete Weber, Angela Cristine Bersch-Ferreira, Rachel Helena Vieira Machado, Camila Ragne Torreglosa, Enilda Maria de Sousa Lara, Lucas Ribeiro da Silva, Renato Hideo Nakagawa Santos, Debora Harumi Kodama Miyada, Erica Regina Ribeiro Sady, Rosana Perim Costa, Leopoldo Piegas, Erlon Oliveira de Abreu-Silva, Alexandre Schaan de Quadros, Camila Weschenfelder, Júlia Lorenzon dos Santos, Gabriela Corrêa Souza, Suena Medeiros Parahiba, Ana Paula Trussardi Fayh, Danielle Soares Bezerra, Ana Paula Perillo Ferreira Carvalho, Malaine Morais Alves Machado, Sandra Mary Lima Vasconcelos, Jéssika Araújo, José Albuquerque de Figueiredo Neto, Luciana Pereira Pinto Dias, Francisca Eugenia Zaina Nagano, Cássia Cristina Paes de Almeida, Annie Seixas Bello Moreira, Débora Pinto Gapanowicz, Eduardo Purgatto, Marcelo Macedo Rogero, Geni Rodrigues Sampaio, Elizabeth Aparecida Ferraz da Silva Torres, Graziela Biude Silva Duarte, Alexandre Biasi Cavalcanti

**Affiliations:** 1 HCor Research Institute (IP-HCor), Hospital do Coração (HCor), Abílio Soares Street, 250, 12th floor, São Paulo, SP Zip Code 04004-050 Brazil; 2 Graduate Program in Health Sciences (Cardiology), Instituto de Cardiologia/Fundação Universitária de Cardiologia (IC/FUC), Porto Alegre, Rio Grande do Sul Brazil; 3 Health Knowledge Implementation Laboratory (LICS), Hospital do Coração (HCor), São Paulo, São Paulo Brazil; 4 Division of Cardiology, Hospital do Coração (HCor), São Paulo, São Paulo Brazil; 5 Hemodynamics Service, Hospital do Servidor Público Estadual (HSPE), São Paulo, São Paulo Brazil; 6 Division of Nutrition, Hospital de Clínicas de Porto Alegre (HCPA), Porto Alegre, Rio Grande do Sul Brazil; 7 Post-Graduation Program in Food, Nutrition and Health, School of Medicine, Universidade Federal do Rio Grande do Sul (UFRGS), Porto Alegre, Rio Grande do Sul Brazil; 8 Department of Nutrition, Universidade Federal do Rio Grande do Norte (UFRN), Natal, Rio Grande do Norte Brazil; 9 Faculty of Health Science of Trairi, Universidade Federal do Rio Grande do Norte (FACISA-UFRN), Santa Cruz, Rio Grande do Norte Brazil; 10 Clinical Nutrition Unit, Hospital de Clínicas, Universidade Federal de Goiás (HC-UFG/EBSERH), Goiânia, Goiás Brazil; 11 Faculty of Nutrition, Universidade Federal de Alagoas (UFAL), Maceió, Alagoas Brazil; 12 Department of Cardiology, Universidade Federal do Maranhão (UFMA), São Luiz, Maranhão Brazil; 13 Complexo Hospital de Clínicas da Universidade Federal do Paraná (HC-UFPR), Curitiba, Paraná Brazil; 14 Universidade Federal do Paraná (UFPR), Curitiba, Paraná Brazil; 15 Instituto Nacional de Cardiologia (INC), Rio de Janeiro, Rio de Janeiro Brazil; 16 Department of Food Science and Experimental Nutrition/Food Research Center, Faculty of Pharmaceutical Sciences, Universidade de São Paulo (USP), São Paulo, São Paulo Brazil; 17 Department of Nutrition, School of Public Health, Universidade de São Paulo (USP), São Paulo, São Paulo Brazil; 18 Department of Food Science and Experimental Nutrition, Faculty of Pharmaceutical Science, Universidade de São Paulo (USP), São Paulo, São Paulo Brazil

**Correction to: Trials 22, 582 (2021)**

**https://doi.org/10.1186/s13063-021-05494-0**

Following the publication of the original article [1], we were notified that Tables 3 and 4 were identical.

Originally, Table 3 was published as a copy of Table 4. This has now been corrected.

Table 4 remains unchanged.

The original article has been corrected.
