# Supplementary material for: Effects of a Brazilian cardioprotective diet and nuts on cardiometabolic parameters after myocardial infarction: study protocol for a randomized controlled clinical trial
Source: Trials. 2021 Sep 1;22:582. doi: 10.1186/s13063-021-05494-0 (PMC8411551; doi:10.1186/s13063-021-05494-0)

**Supplementary Material**

**Table S1.** Food group (portions/day)^a,b^ standard harmonization according to energy requirement ranges for DICA Br prescription.

| BALANCE Trial color  classification  Energy  requirements (kcal/d) | 1,400 | 1,600 | 1,800 | 2,000 | 2,200 | 2,400 |
| --- | --- | --- | --- | --- | --- | --- |
| Green^c^ | 9 | 11 | 11 | 12 | 14 | 16 |
| Yellow^d^ | 6 | 7 | 9 | 10 | 11 | 13 |
| Blue^e^ | 2 | 2 | 3 | 3 | 4 | 4 |
| Red^f^ | 0 | 0 | 0 | 0 | 0 | 0 |

BALANCE: Brazilian Cardioprotective Nutritional Program.

^a^This content was published in the BALANCE research protocol, reproduced with permission and available upon request for the authors; ^b^Participants were not instructed to aim for a number of certain types of foods within a food group (green, yellow or blue), and a specific counseling about fluids intake was not given; ^c^Types of foods belonging to green group: fruits, vegetables, beans, skinny milk and yogurt; ^d^Types of foods belonging to yellow group: cereals, breads, nuts, oils; ^e^Types of foods belonging to blue group: eggs, meat, fish, chicken, cheese; ^f^Types of foods belonging to red group: ultra-processed foods.

**Chart S2.** Example of a menu/daily foods distribution and corresponding number of colored food groups as a ludic strategy for improving compliance with DICA Br prescription.

| **Meal/foods** | **Homemade measure** | **DICA Br food group** |
| --- | --- | --- |
| *Breakfast* |  | 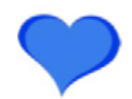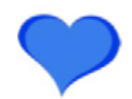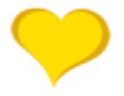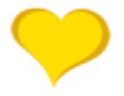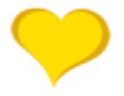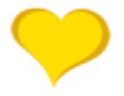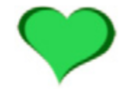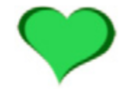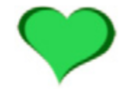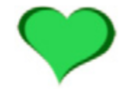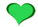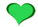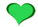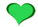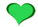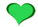 |
| Bread | 1 slice | 1 Yellow |
| Cream cheese | 2 teaspoons | 1 Blue |
| Orange juice | 1 glass | 1 Green |
| Papaya | 1/2 unit | 1 Green |
| Fat free skim milk | 1 cup | 1 Green |
|  |  |  |
| *Lunch* |  |  |
| Salad | 1/4 plate | 1 Green |
| White rice | 1 tablespoon | 1 Yellow |
| Cabbage | 1 tablespoon | 1 Green |
| Steak | 80g | 1 Blue |
| Boiled potatoes | 2 tablespoons | 2 Yellow |
| Orange fruit | 1 unit^a^ | 1 Green |
|  |  |  |
| *Snack* |  |  |
| Fat free yogurt | 1 unit^b^ | 1 Green |
| Banana | 1 unit^c^ | 1 Green |
|  |  |  |
| *Dinner* |  |  |
| Bread | 1 slice | 1 Yellow |
| Vegetable soup | 1 soup plate | 1 Green |
| Peach | 1 unit^d^ | 1 Green |

^a^1 unit = 180g; ^b^1 unit = 200g; ^c^1 unit = 70g; ^d^1 unit = 60g.

**Table S2.** Centesimal composition, fatty acid profile, and minerals´ content of the nuts provided in DICA-NUTS study.

|  | Peanuts  (100g) | Brazil nuts  (100g) | Cashew  (100g) | Mixed nuts*  (100g) | DICA-NUTS Mix (30g) |
| --- | --- | --- | --- | --- | --- |
| Energy (Kcal) | 610.3 | 705.1 | 610.9 | 649.8 | 194.9 |
| Carbohydrate (g) | 16.1 | 8 | 21.8 | 14 | 4.2 |
| Protein (g) | 25.1 | 15.5 | 22.7 | 24.7 | 7.4 |
| Total fat (g) | 49.5 | 67.9 | 48.1 | 55 | 16.5 |
| Saturated fatty acids (g) | 7.3 | 17.2 | 9.2 | 11.1 | 3.3 |
| Palmitic acid (C16:0) | 4.1 | 9.2 | 6.3 | 6.6 | 2 |
| Stearic acid (C18:0) | 1.0 | 7.5 | 3.0 | 3.5 | 1.1 |
| Arachidic (eicosanoic) acid (C20:0) | 0.7 | - | - | 0.3 | 0.1 |
| Behenic (docosanoic) acid (C22:0) | 0.9 | 0.1 | - | 0.3 | 0.1 |
| Lignoceric acid (C24:0) | 0.6 | 0.3 | - | 0.4 | 0.1 |
| Monounsaturated fatty acids (g) | 40.4 | 20.4 | 30.5 | 31.6 | 9.5 |
| Palmitoleic acid (C16:1) | - | 0.2 | - | 0.1 | - |
| Oleic acid (C18:1 n9c) | 38.3 | 19.4 | 29.9 | 30 | 9 |
| Nervonic acid (C24:1) | 2.1 | 0.8 | 0.7 | 1.4 | 0.4 |
| Polyunsaturated fatty acids (g) | 1.8 | 30.4 | 8.4 | 13.5 | 4.1 |
| Linoleic acid (C18:2 n6c) | 1.4 | 30.2 | 8.1 | 13.2 | 4 |
| Linolenic acid (C18:3 n3) | 0.4 | 0.2 | 0.2 | 0.3 | 0.1 |
| Insoluble dietary fiber (g) | 8.3 | 7.6 | 4.7 | 5.2 | 1.6 |
| Soluble dietary fiber (g) | 1.0 | 1.0 | 2.8 | 1.1 | 0.3 |
| Total dietary fiber (g) | 9.3 | 8.6 | 7.4 | 6.3 | 1.9 |
| Humidity (%) | 4.7 | 2.8 | 4.3 | 4.3 | 1.3 |
| Ashes (%) | 2.0 | 3.6 | 2.3 | 2.8 | 0.8 |
| Barium (mg) | < DL | 5.6 | < DL | 7.2 | 2.16 |
| Calcium (mg) | 110.9 | 219.2 | 71.2 | 162.7 | 48.8 |
| Copper (mg) | < DL | < DL | 0.27 | 0.08 | 0.024 |
| Phosphorus (mg) | 57.5 | 2.5 | 15 | 57.5 | 17.3 |
| Magnesium (mg) | 270.9 | 358.1 | 279.5 | 352.3 | 105.7 |
| Manganese (mg) | 1.6 | 1.07 | 1.6 | 1.1 | 0.34 |
| Potassium (mg) | 538.7 | 474.7 | 506.7 | 562.7 | 168.8 |
| Selenium (µg) | 638 | 602 | 707 | 731 | 219.3 |
| Sodium (mg) | 16 | 10.7 | 13.3 | 10.7 | 3.2 |
| Zinc (mg) | 18.5 | 4.1 | 5.04 | 40.3 | 12.1 |

* Approximately 33% of each nut was used to compose the mix for chemical analyses.

< DL: value below the detection limit.

**Appendix I**

Methodology used for chemical analysis in all nuts offered in DICA-NUTS Trial.

*1. Determination of humidity*

Approximately 10 g of homogenized sample was weighed in a tared porcelain capsule. The capsule was transferred to a vacuum oven at 105 ° C, where the material was desiccated until a constant weight was obtained.^1^

*2. Determination of ashes*

2 g of desiccated sample were weighed in a calcined and tared porcelain crucible. Carbonization was started on a heating plate, followed by transfer to a muffle furnace at 550° C for 8 hours. After cooling, the crucible was transferred to a desiccator and weighed as soon as it reached room temperature. This procedure was repeated until constant weight was obtained.^2^

*3. Determination of lipids*

2 g of the dry sample were weighed and transferred to a treated cellulose cartridge. Lipid extraction was performed with anhydrous ethyl ether for approximately 5 h in the Soxhlet apparatus. After the time, the flask containing the lipids was disconnected from the device and kept open on a heating plate at 40o C in a chapel until the organic solvent evaporated. The flask was dried in an oven at 105° C until the solvent was completely evaporated, and residual moisture was removed. Next, it was cooled in a desiccator and weighed. The procedure was repeated until constant weight was obtained. The dry and defatted residue was kept in a desiccator for further analysis.^3^

*4. Determination of proteins*

Approximately 60 ±2 mg of dry and defatted sample, 1.9 ± 0.1 g of K2SO4, 50 ±10 mg of CuSO4 (used as a catalyst) and 2.5 ±0.1 ml of concentrated H2SO4 were added in an appropriate test tube for hot digestion. A blank test was also prepared and, together with the sample, was digested at 350-400° C for 3 hours in a digester block. At the end, 5 mL of distilled water was added to the digested sample.

For distillation, the tube test was coupled to the Kjeldahl distiller, with 10 mL of 60% NaOH being added. The distillate was collected in a 125 mL erlenmeyer flask containing 5 mL of saturated boric acid solution and 4 drops of the indicator (methyl red + methylene blue), forming ammonium borate.

The distillation titration was performed with factorized 0.02N HCl. Based on the equivalence between the nitrogen content and the spent HCl 0.02 volume, the protein content was calculated using the factor 6.25.^4^

*5. Determination of dietary fibers*

Dietary fiber content was determined according to the AOAC 991.43 method,^5^ with separation of insoluble and soluble fractions, using the Total Dietary Fiber Assay Kit (Megazyme K-TDFR) enzyme kit. The determination was performed in quadruplicate and the total dietary fiber content was calculated by the sum of insoluble and soluble fractions.

The samples were subjected to sequential enzymatic hydrolysis with thermo-resistant α-amylase (98-100° C, pH 8.2, 30 min), protease (60° C, pH 8.0, 30 min) and amyloglycosidase (60° C, pH 4.5, 30 min) for removing starch and protein. For insoluble fraction determination, the solution resulting from hydrolysis was filtered and the residue washed with hot water, 78% ethanol and 95% acetone. For soluble fraction determination, the filtered solution was added with heated ethanol (60° C) until reaching the final concentration of 78%. After 1 hour, the solution was filtered and washed with 78% ethanol and 95% acetone. All filter crucibles were dried and weighed. Values of insoluble and soluble dietary fibers residues were corrected by analyzing protein and ashes using the same methods described before.

*6. Determination of fatty acid methyl esters*

Fatty acids were determined by saponification of aliquots of lipid extract according to the method of Metcalfe et al.^6^ For this, a 100 mg aliquot of lipids was taken, then 4 mL of methanolic NaOH (0.5 N) was added to the sample. After this procedure, the sample was homogenized and kept in a water bath at 100o C for 2 minutes. After immediate cooling, 3 mL of boron-methanol trifluoride was added to the sample, stirred lightly and again the sample was taken to the water bath at 100o C for another 2 minutes. Then, in the immediately cooled sample, 3 ml of saturated sodium chloride solution (NaCl) were added, the tubes were shaken slightly and 3 ml of hexane (HPLC grade) were added; the upper phase was separated and again 3 mL of hexane (HPLC grade) was added, separating the upper phase once more, which was then evaporated in a nitrogen atmosphere to an approximate volume of 1 mL, which was stored (–26º C) in vials model 0221-34 274-91 with cover and septum (SHIMADZU).

The fatty acid profile was determined in a gas chromatograph SHIMADZU, CG-2010 model, equipped with a capillary column of fused silica (15m x 0.1 mm, 0.1 µm, DB-FFAP, J&W Scientific, Agilent), and hydrogen was used as a carrier gas with a flow rate of 1.5 mL/min. The injector and detector temperatures were 250oC and 260oC, respectively. The temperature programming of the column was initial 140° C with an increase of 4° C/min, until reaching a plateau of 240° C, remaining at this temperature for 15 minutes. The split ratio used in the injector was 150.

The standard used was a mixture of thirty-seven fatty acid methyl esters - 37, code 47885 (Sigma Chemical Co). The volume of injection was 1 mL, using an automatic AOC 20i injector. The results were expressed as % of the total fatty acids present in the sample.

*7. Determination of available carbohydrates and energy*

Available carbohydrates of each nuts were determined by the difference between 100 and the sum of proteins, lipids and total fibers. For the mix, carbohydrates were determined as a proportion (30%) of all nuts’ carbohydrate average. Energy content for each nut was determined by the Atwater method^7^, and for the mix was made a proportion of all nuts’ energy average.

*8. Determination of mineral´s content*

Samples were opened in a microwave oven (CEM, model: MarsXpress), in which 0.20g in 50ml were used for phosphorus evaluation and 0.75g in 200ml were used for the other elements. For selenium determination, total radiation-assisted digestion in HNO_3_ + H_2_O_2_ technique was used.

The methods used for detection were:

- Barium, Calcium, Copper, Magnesium, Manganese and Zinc: atomic absorption spectrophotometry;

- Sodium and Potassium: flame photometry;

- Phosphorus: colorimetry;

- Selenium: generation of hydrides (model: VGA 77, Agilent Technologies) coupled to an absorption spectrometer atomic flame (FAAS) (model: AA 240FS, Agilent Technologies).

References:

1. Bradley RL. Moisture and total solids analysis. In: Nielsen SS. Introduction to chemical analysis of foods, Boston, Jones & Bartlett, 1994. p.93-111.

2. Harbers LH. Ash analysis. In: Nielsen SS. Introduction to chemical analysis of foods, Boston, Jones & Bartlett, 1994. p.113-121.

3. Min DB. Crude fat analysis. In: Nielsen SS. Introduction to chemical analysis of foods, Boston, Jones & Bartlett, 1994. p.181-205.

4. Chang SKC. Protein analysis. In: Nielsen SS. (ed.). Food analysis. 2nd ed. Mariland. Chapman and Hall Food Science. 1998. p. 237-249.

5. Lee SC et al. Determination of total, soluble and insoluble dietary fiber in food enzymatic, gravimetric method, MES-TRIS buffer: collaborative study. J. Assoc. Off. Anal. Chem. 1992; 75:395-416.

6. Metcalfe et al. Rapid preparation of fatty acid esters from lipids for gas chromatographic analysis. Analytical Chemistry. 1966;12:514.

7. Atwater WO, Bryant AP. The availability and fuel value of food materials. Washington, DC: US Government Printing Office, 1900 (Agriculture Experiment Station 12th Annual Report 73-110).

**Appendix II**

DICA-NUTS Trial Consent Form.

Consent form

You are being invited to participate in the study entitled “*Brazilian Cardioprotective Diet and Nuts in Post-acute Myocardial Infarction (DICA-NUTS)*” *funded by* Heart Hospital – Syrian Beneficent Society (Hcor) and the Brazilian Ministry of health. Before your consent, it is important that you understand the main objectives of this research and clarify all your doubts. Your participation in this study is absolutely voluntary, and in case you choose to take part in this study, you or your legal party will need to sign and initial two copies of this document. You will be able to keep one copy of this document, and the other will be kept by the researcher, who will sign and initial the document as well. Please, read all the information below carefully and ask any questions you may have. You have the right to discuss this document with your family, friends or doctor, before deciding whether to take part or not.

You have the right to refuse participation in this study. If you decide to terminate your participation after the study has begun, you may do so at any given moment, we just ask you to let the researchers know of your decision as soon as possible. One last appointed may be scheduled with the researchers to answer your doubts before terminating your participation in the study.

In this Project, we intend to assess the effect of a diet adapted for Brazilian eating habits with or without nuts (which are foods typically consumed in Mediterranean European countries) on the blood levels of fats (total cholesterol, LDL cholesterol [bad], HDL cholesterol [good] and triglycerides), and sugars (glucose, glycated hemoglobin and insulin), on your weight and measurements of waist and hip circumferences. We will also assess some molecules in your blood that are related to your risk of developing cardiovascular diseases. They are called adipokines, inflammatory biomarkers and others that associate to the processing of aging.

It is important that we evaluate these items because heart diseases are the main cause of death in Brazil and worldwide. Healthy foods may contribute to the reduction of some of these diseases, such as infarction and strokes. The best type of diet to prevent and treat these conditions is the Mediterranean diet. However, most of the foods recommended by it are not usually found in Brazil at affordable prices. Additionally, some studies suggest that the frequent intake of nuts can help in the treatment of cardiovascular diseases. That is why we intend to test this Brazilian diet associated to the nuts we usually eat here.

1. **How many participants are there in the study and how long does it last?**

We hope to include 500 participants with more than 40 years old and previous myocardial infarction, in at least 2 regions of Brazil. Your participation in the study will last 4 months.

1. **Which are the study’s interventions?**

After you consent to your participation, you will be randomly assigned to either of two groups. Nor you or the researcher will be able to choose which group you will be assigned to.

- If you are assigned to group 1: you will be followed by a registered dietitian (RD) who will provide you with nutritional counseling based on the cardioprotective Brazilian Diet and on the premises of the Brazilian Society of Cardiology to stimulate your cardiovascular health. You will be followed for 4 months.
- If you are assigned to group 2: you will be followed by a registered dietitian (RD) who will provide you with the same nutritional counseling mentioned above, added with a daily amount (30g/day) of mixed nuts (composed by peanuts, cashew nuts and Brazilian nuts) . You will also be followed for 4 months.

1. **What are the study procedures?**

During the 4 months of follow up, you will be asked to schedule 5 appointments with the research team: first visit and within 30 days, 60 days, 90 days and the last visit will be at 120 days. In every appointment we will measure your weight, height, waist and hip circumferences with a measurement tape and scale. We will also ask you about your overall health, physical activity patterns, and your eating habits in the past few months, as well as in the day before your first visit. We will ask you to provide blood samples in the first and last appointment. We will measure the fat and sugar levels in the blood of all participants, but will group draw those who will undergo the anti-aging molecules after the study is completed. This will be possible because we will identify your blood sample with the code you will receive after your first study visit. All appointments will take place in private rooms in the outpatient service you were recruited.

Throughout the 4 months of follow up, you may receive phone calls, cell phone messages or emails from our team of researchers, to remind you of your next appointments, to ask you about your general health or to stimulate you to maintain healthy habits.

1. **What will I have to do?**

You will need to come to the scheduled appointments for the next 4 months, every 30 days. It is important that you do not miss these appointments so that the RDs can check your dietary progress and collect all data necessary to the study.

1. **Does this study involve any risks?**

You will be exposed to minimum risk, since we are only asking you to fill in questionnaires, provide two blood samples and keep a healthy diet. In case you do not feel comfortable or feel tired while answering the questions in the forms, you can stop at any moment you choose. As for the blood collection, the risks are the same as any other routine blood exam, and you may experience bruises and soft pain in the arm which you decide to give blood. All materials used for blood collection will be sterile and you may use cold ice packs locally.

If you are assigned to group 2, the intake of nuts is usually safe if you do not have any known food allergies. If you do, it is important to tell the researchers. However, there may be cases of nausea, stomach ache, diarrhea, itches or symptoms of allergy, and feeling of bloating. In case you feel any of these symptoms, the RD will advise the correct procedure according to what you report (example: you may be asked to bake your nuts instead of eating them plain, or even stop eating them until you feel better). If you still do not adapt to the mixed nuts, you can stop eating them at any point. In this case, you will remain being followed until the end of the study period. Please tell the RD what you are feeling as soon as possible.

1. **What are the benefits of this study?**

Studies have shown that a healthy diet associated or not to the regular intake of nuts improves your cardiovascular health, as well as you lipid and sugar profiles in your blood, your weight and body composition. Because of that, either way you will be able to improve your health, regardless of the group you are assigned to. We also hope the data we collect from your reports will contribute to science and the way treat patients with myocardial infarction, and help many other people in the future.

1. **Will there be payment if I take part in this study?**

No. You will not be paid to participate in our project. However, you will be compensated for the transportation costs in all 5 visits of the study.

1. **Who will have access to my personal information?**

All data collected in this research will be confidential and only the research team will have access to it. The information will be used only for research purposes. The results of this project will be released without your personal identification and all data will be anonymously handled, as you will be identified with a number in your first visit.

This study is expected to end in 2021. However, the blood we do not analyze in this period will be stored in a freezer specially allocated for that at our HCor. We may use these samples to make new analysis (for example, genetic components of blood) in partnership with other Brazilian institutions or international ones. Hence, if you consent to participate in this study you will also consent that we store your blood samples in our biorepository. If we do test your stored blood to perform analysis on the anti-aging molecules we mentioned before, we guarantee they will be performed with quality and anonymously. If we decide to perform any other analysis not mentioned in this document, we will ask for your consent once again.

**9) Who should I contact in case of doubts?**

This study was approved by the Ethics Committee of Hcor, a group that revised all studies in our institution and protects your rights as a participant. If you have any doubts about the ethical or safety aspects of the study, please contact them at: 11 3886 4688 or in person at Abrão Dib Street, 50 – Paraíso – São Paulo/SP. If you prefere email: [etica.pesquisa@hcor.com.br](mailto:dvsilva@hcor.com.br). Working hours: Monday to Friday, 8am to 5pm. The research team is also available to answer any of your questions at any moment of the study: Aline Marcadenti – Principal investigador: 11 30536611- R: 3558 (Address: Abrão Dib Street, 250 – 12th floor - Paraíso – São Paulo/SP). If we find any new information that may interfere with your decision to participate in this study, we will ask for your re-consenting as soon as possible.

**Declaration of consent**

I agree to take part in the study entitled “*Brazilian Cardioprotective Diet and Nuts in Post-acute Myocardial Infarction (DICA-NUTS)*”, under the responsibility of Aline Marcadenti.

I allow the use of the information I provide, exclusively for this research purposes.

I am aware of all the procedures I will undergo and of the possible minimum risks I will be exposed to.

I can ask for further information about the study activities at any moment and will be promptly answered by the researchers.

| I was given one copy of this document. |  |  |  | Finger print (IF necessary) |
| --- | --- | --- | --- | --- |
| Name of participant  Date*: _____/_____/_______ |  | Signature |  |  |
| Name of participant legal party  Data*: _____/_____/_______ |  | Signature |  | Specify relation with participant |
| Name of impartial witness ****  Date: _____/_____/_______ |  | Signature |  |  |
|  |  |  |  | ( ) Investigator  ( ) Coordinator  ( ) Data collection  ( ) Nurse |
| Name of person who explained this document  Date*: _____/_____/_______ |  | Signature |  | Delegation in the study |
| ** may be necessary in cases of physical disabilities (i.e blind or illiterate persons). The witness must be present throughout the entire explanation of the document. | | | | |

**Appendix III**

Biorepository: protocol for plasma separation from whole blood and sampling storage.

All participants in this trial will be asked to provide blood samples at their first and final visit. Most of the sample will be used to assess primary and secondary outcomes, and part of the sample will be processed and stored to for the biorepository. Researchers will be trained to process the samples for storage according to ICH/GCP guidelines and follow proper standard operating procedures (SOPs). We describe the related procedures as follows:


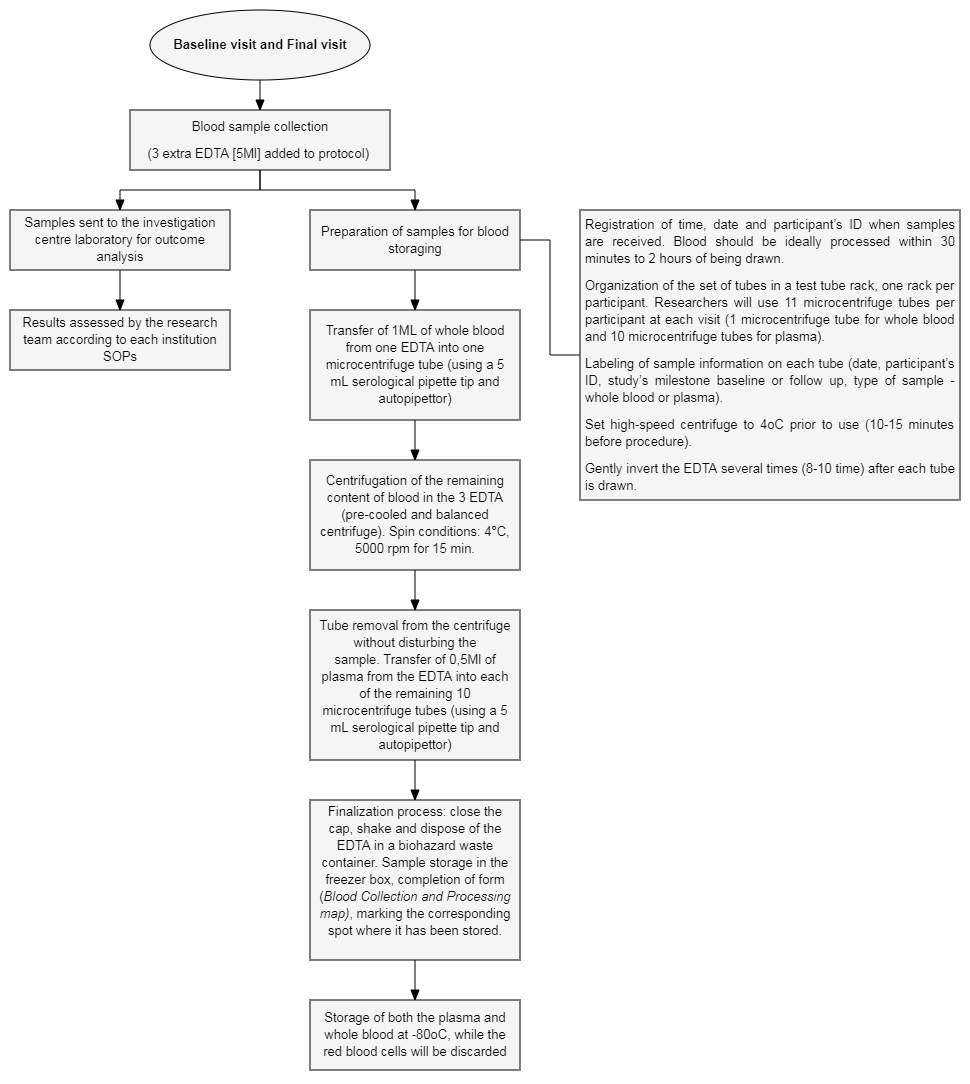

Supplement: Supplementary file 1 — Additional file 1. [file 13063_2021_5494_MOESM1_ESM.doc]
